# A Facile Nanoparticle Immunoassay for Cancer Biomarker Discovery

**DOI:** 10.1186/1477-3155-9-20

**Published:** 2011-05-23

**Authors:** Qun Huo, Jimmie Colon, Adam Cordero, Jelena Bogdanovic, Cheryl H Baker, Steven Goodison, Marianna Y Pensky

**Affiliations:** 1 NanoScience Technology Center and Department of Chemistry, University of Central Florida, 12424 Research Parkway Suite 400, Orlando, FL 32826, USA; 2 MD Anderson Cancer Center Orlando, Cancer Research Institute, 6900 Lake Nona Blvd, Orlando, FL 32827, USA; 3 Department of Mathematics, University of Central Florida, 4000 Central Florida Blvd, Orlando, FL 32816, USA

## Abstract

**Background:**

Gold nanoparticles (AuNPs) scatter light intensely at or near their surface plasmon wavelength region. Using AuNPs coupled with dynamic light scattering (DLS) detection, we developed a facile nanoparticle immunoassay for serum protein biomarker detection and analysis. A serum sample was first mixed with a citrate-protected AuNP solution. Proteins from the serum were adsorbed to the AuNPs to form a protein corona on the nanoparticle surface. An antibody solution was then added to the assay solution to analyze the target proteins of interest that are present in the protein corona. The protein corona formation and the subsequent binding of antibody to the target proteins in the protein corona were detected by DLS.

**Results:**

Using this simple assay, we discovered multiple molecular aberrations associated with prostate cancer from both mice and human blood serum samples. From the mice serum study, we observed difference in the size of the protein corona and mouse IgG level between different mice groups (i.e., mice with aggressive or less aggressive prostate cancer, and normal healthy controls). Furthermore, it was found from both the mice model and the human serum sample study that the level of vascular endothelial growth factor (VEGF, a protein that is associated with tumor angiogenesis) adsorbed to the AuNPs is decreased in cancer samples compared to non-cancerous or less malignant cancer samples.

**Conclusion:**

The molecular aberrations observed from this study may become new biomarkers for prostate cancer detection. The nanoparticle immunoassay reported here can be used as a convenient and general tool to screen and analyze serum proteins and to discover new biomarkers associated with cancer and other human diseases.

## Background

Gold nanoparticles (AuNPs) scatter light intensely at or near the surface plasmon resonance wavelength region [1,2]. By combining the exceptional light scattering property of AuNPs with dynamic light scattering, we recently developed a new assay technique, NanoDLSay™, for bimolecular detection and analysis [3-9]. Several other groups have demonstrated the combined use of AuNPs and DLS for quantitative estimation of nanoshells in whole blood [10], highly sensitive detection of small biological molecules [11], toxic metal ions and explosives [12-15]. Although DLS has not been traditionally used for quantitative analysis, these recent studies by different groups demonstrate that DLS can be used as a rather reliable and sensitive technique for quantitative detection and analysis of chemical and biological species.

**Figure 1:**
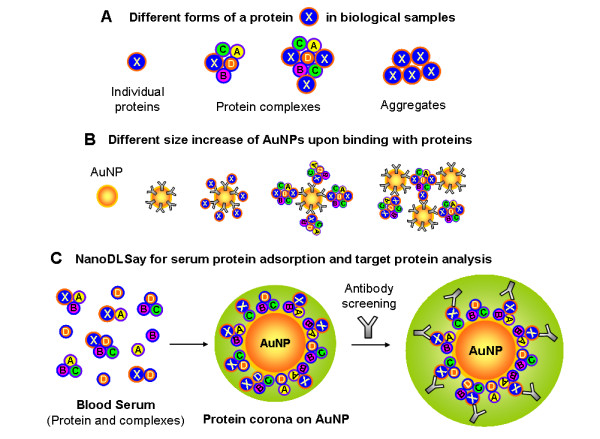
**Illustration of the principle of NanoDLSay used for serum protein detection and analysis**. (A) Illustration of proteins in different forms. X refers to a target protein, while A, B, C, D, etc. refer to any known or unknown biomolecules that are complexed to the target protein X. (B) Different particle size increase caused by the adsorption or binding of proteins in different forms. (C) The principle of the assay used in the present study: In the first step of the assay, a serum sample is mixed with a citrate-protected AuNP solution. After the formation of a stable protein corona around the AuNPs, an antibody specific to the interested target protein is added to the assay solution. When a target protein is present on the nanoparticle surface, a further size increase of the nanoparticles can be observed through DLS measurement.

DLS is an analytical technique that is used routinely for particle size analysis [16]. Current DLS instruments can detect particle size differences of 1-2 nm. Proteins are biomacromolecules with a hydrodynamic diameter of a few nanometers: for example, the hydrodynamic diameter of a bovine serum albumin is about 5-6 nm, and the hydrodynamic diameter of an IgG molecule is around 7-10 nm. When a layer of protein molecules are adsorbed or bound specifically to a gold nanoparticle surface, the hydrodynamic diameter of the nanoparticle-protein complex will increase by as much as twice of the diameter of the protein [5,6]. The most unique capability of NanoDLSay™ is that the assay can directly reveal the complexing status of target proteins. In biological systems, a protein can exist in three different forms as illustrated in Figure 1A: as individual molecules; in complexes with other proteins or biomolecules; and in aggregates. When a protein, protein complex or aggregate is adsorbed or bound to AuNPs, this will cause different size changes of the AuNP probes as shown in Figure 1B, and such differences can be readily detected by DLS [7-9]. In a previous study, we observed that a prostate cancer biomarker, prostatic acid phosphatase (PAP) is substantially more complexed/aggregated in prostate cancer tissue than tissues with normal and benign conditions [8]. Normal and non-cancerous benign prostate conditions can be distinguished from prostate cancer based on the aggregation level of PAP in tissue samples. Luchter-Wasylewska et al [17,18]. showed that at elevated concentration, PAP tends to form oligomers and aggregates. Our finding suggests for the first time that the complexing/aggregation status of a protein instead of its concentration change may potentially serve as a new type of cancer biomarker. Using NanoDLSay™, we have further discovered a new heteromeric protein complex that is formed between epidermal growth factor receptor (EGFR), Src and Stat3 in pancreatic cancer cells [9].

Recently, Dobrovolskaia et al. reported a systematic study on human plasma protein adsorption to AuNPs [19]. This work demonstrated that when citrate-protected AuNPs are mixed with blood plasma, proteins from the plasma adsorb to the AuNPs to form a "protein corona". They identified more than 60 proteins in the protein corona through a 2D PAGE gel isolation followed by a Mass Spectroscopy analysis. Based on this previous work, we raised the question: would there be any differences in the size and composition of the protein corona between cancerous and non-cancerous serum samples. If so, such molecular differences could potentially be used as a new type of serum biomarker for cancer detection and diagnosis. To answer this question, we designed an assay format as illustrated in Figure 1C based on the principle of NanoDLSay™: A serum sample is first mixed with a citrate-protected AuNP solution. After a protein corona is formed on the nanoparticle surface, an antibody for a suspected target protein is added to the assay solution. If the target protein is present in the protein corona, the binding of the antibody to the protein corona will cause a further increase of the average nanoparticle size; otherwise, the nanoparticle size will remain unchanged.

Using this simple assay, we analyzed the serum samples collected from mice models and human donors with and without prostate cancer. Indeed, we observed a number of interesting molecular differences in the adsorbed protein corona between cancer and non-cancerous samples. Although we tested only a few target proteins in the present study, it is easy to see that this assay may be used as a general approach to analyze other target proteins and to discover potential biomarkers for the detection, diagnosis and prognosis of diseases.

## Results

To have a complete understanding of our new assay and the assay results, several important technical details about the assay warrant explanation. First, the exceptionally strong light scattering of AuNPs is essential for the successful application of NanoDLSay™ for protein detection and analysis in blood serum samples. Blood serum is a rather complicated biological fluid: it contains a large amount of proteins, protein complexes, aggregates, colloidal particles and other biomacromolecules or biomacromolecular assemblies that also scatter light intensely. As an optical probe based on light scattering detection, the scattered light intensity from the AuNPs must be substantially stronger than the scattered light from the biological samples to avoid the background interference from the sample matrix. The AuNP solution used in the present study has a hydrodynamic diameter of 90 nm and at a concentration of 10 pM, it can generate a scattered light intensity of around 400 kcps (kilo counts per second) at a laser power of 0.04 mW. At this low laser power, the scattered light from the serum samples added to the AuNP solution is below the reliable detection limit of the DLS instrument. This means that the nanoparticle size increase observed from the assay solutions through DLS analysis is solely caused by protein adsorption or binding to the AuNPs. Other biomacromolecules or colloidal particles from the serum samples that are not attached to the AuNPs will not contribute to the DLS signal. In the subsequent antibody screening of target proteins, a high concentration of antibody is added to the assay solution to bind with target proteins that remain in solution or in the protein corona. Because the binding of the antibody with the target proteins that remain in solution does not contribute to the size change of the AuNP probes, there is no need to separate the AuNPs from the assay solution to detect the target proteins that are adsorbed to the AuNPs. Without a physical separation step, the binding event that occurs on the AuNPs is isolated from the rest of the assay solution and is selectively detected by DLS measurement.

A second important detail is the relative ratio of the AuNPs and proteins involved in the assay. The concentration of AuNP solution used in this study is 10 pM. The total protein concentration of a human serum sample is typically in the high 10s to 100 mg/mL range, corresponding to a molar concentration in the 10s to 100 of μM range. In the assay solution (2 μL serum was mixed with 40 μL AuNP solution), the total protein concentration is 10^5^-10^6 ^times of the AuNPs. Even after 10-fold dilution, the serum protein concentration is still about 10^4^-10^5 ^times of the AuNP concentration. In other words, in the assay solution, serum proteins are in substantially large excess over the AuNPs. This large excess of serum proteins versus AuNPs is to ensure a saturated adsorption of serum proteins on the AuNPs. Only under the saturated adsorption condition, the particle size increase of the assay solution can be used to deduce the hydrodynamic diameter of the proteins adsorbed on AuNPs.

A third important detail is the low concentration of the AuNP solution used in the assay, 10 pM. This low concentration of AuNP was used to avoid nanoparticle cluster formation caused by protein interaction. If substantial nanoparticle clusters are formed in the assay solution, than the average particle size increase of the assay solution cannot be used to deduce the protein size. Initially, we used a 40 nm citrate-protected AuNP with a concentration of 1 nM for the assay. When serum samples were mixed with this AuNP solution, significant nanoparticle clustering was observed, judging from the broad and multi-model particle size distribution curves. By using the 90 nm AuNPs at a concentration of 10 pM, the particle size distribution curves of all assay solutions remained narrow and monodispersed (polydispersity index around or below 0.2-0.3), indicating that nanoparticle cluster formation during the assay was minimized. In this case, the observed particle size increase of the assay solution can be correlated to the size information of the proteins or protein complexes that were adsorbed to the AuNPs.

### Serum protein adsorption to AuNPs from mice with prostate tumor and healthy controls

Three orthotopically injected mouse models were prepared for this study: one with a fast growing prostate cancer cell line PC3; one with a slow growing tumor cell line LnCaP; and a third group of mice injected with PBS saline solution as control (See the Additional File 1 for details on mice model development, tumor examination and serum sample collection) [20-22]. The size and the weight of each tumor are summarized in Table 1. As expected, mice injected with PC3 cells grew substantially larger tumors (in the gram range) than those injected with LnCaP cells (in the 10s to 100 mg range). No tumor was found in the prostate of control mice. The average weight ratio of tumor over the body weight is approximately 5% for the PC3 mice, and less than 0.3% for the LnCaP mice. These ratios would correspond to a tumor weight of 2.5 Kg and 150 g respectively in a human patient with a body weight of 50 Kg.

**Table 1:** Information on the mice models used in the current study

**Mice No**.	Cell Line	Tumor weight(g)	**Tumor Volume (mm**^**3**^**)**	Body weight(g)	Collection date
C 1	n/a	0	0	37.86	5 weeks
C 2	n/a	0	0	39.65	5 weeks
C 3	n/a	0	0	26.77	5 weeks
C 4	n/a	0	0	24.08	5 weeks
C 5	n/a	0	0	28.83	5 weeks
M 1	PC3	1.97	1798	37.71	4 weeks
M 2	PC3	2.37	1280	38.16	4 weeks
M 6	PC3	0.95	1088	37.75	4 weeks
M 7	PC3	0.89	1488	32.13	4 weeks
M 10	PC3	2.92	1987	40.87	4 weeks
M 225	LnCap	0.102	169	31.33	5 weeks
M 999	LnCap	0.072	212	31.68	5 weeks
M 211	LnCap	0.087	365	31.17	5 weeks
M 213	LnCap	0.068	t.s.t.m*	29.31	5 weeks
M 216	LnCap	0.033	t.s.t.m	29.29	5 weeks
M 220	LnCap	0.043	t.s.t.m	33.38	5 weeks

*t.s.t.m.: too small to measure

**Figure 2:**
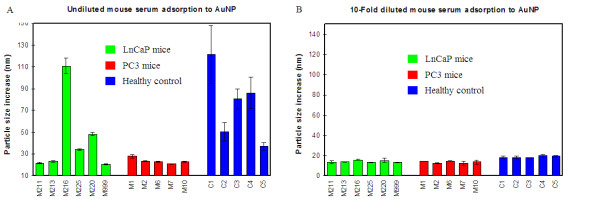
**Mouse serum protein adsorption study on AuNPs**. (A) Undiluted serum; (B) 10-fold diluted serum in 10 mM PB. Particle size increase shown in the graph is the difference between the measured particle size at 8 min (for undiluted serum samples), or 5 min (for diluted serum samples) and the particle size at 1 min after mixing 2 μL of serum with 40 μL of AuNP solution. Error bars are standard deviations from two replicate assays.

We first analyzed the size change of the AuNPs caused by serum protein adsorption. From these analyses, several differences amongst the PC3 mice, LnCaP mice and healthy controls were observed. First, the average particle size increase of the healthy control group was 75 nm, significantly higher than the PC3 mice that had an average particle size increase of 24 nm (Figure 2A). The average particle size increase for LnCaP mice was 43 nm. It was also noticed that within the healthy control or LnCaP mice group, there were substantial variations between individual mice: the particle size increase varied from 20-110 nm for the LnCaP mice, and 40-120 nm for the healthy control mice. As to the PC3 mice, the particle size increase was uniformly low: in the range of 20-25 nm. When the serum samples were first diluted 10-fold, the average particle size increase caused by protein adsorption decreased to 18 nm for the healthy control group, and 15 nm for both the PC3 and LnCaP mice (Figure 2B). These particle size increases were reached after 2-3 minutes following sample-AuNP mixing, and remained unchanged thereafter.

### Target protein detection and analysis from mouse serum-adsorbed AuNPs

After serum protein adsorption on the AuNPs, we then analyzed two target proteins in the protein corona by adding corresponding antibodies to the assay solution: one is IgG and another one is vascular endothelial growth factor (VEGF). IgG is one of the most abundant serum proteins, with a concentration in the 10s mg/mL range. It is reasonable to believe that IgG should be found in the serum protein corona. The target protein analysis was conducted using 10-fold diluted serum samples instead of the undiluted serum samples: because the protein corona formed on the AuNPs has a similar size of 15-18 nm for all three mice groups, the size increase observed from anti-IgG assay can be used directly to compare the IgG levels of different samples.

**Figure 3:**
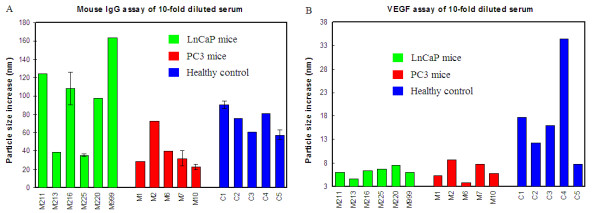
**Mouse IgG (A) and VEGF (B) assay of 10-fold diluted mouse serum adsorbed to AuNPs**. The particle size change presented in both graphs is the difference between the size measured at 5 min after antibody addition and the size just before antibody addition.

Data shown in Figure 3A is the measured particle size difference before and after the addition of anti-mouse IgG antibody to the serum-AuNP mixture solution. The particle size was increased for all mice serum samples, however, LnCaP mice appeared to have a higher level of IgG in the protein corona than the other two groups. Furthermore, there were significant variations in the IgG level of different mice within the same group, particularly in the LnCaP mice group.

Vascular endothelial growth factor (VEGF) is a protein biomarker that is associated with almost all types of cancer due to its essential role in tumor angiogenesis [23-25]. Naturally, we selected VEGF as a target protein to analyze in this study. Rabbit polyclonal anti-VEGF (ab9570) was used for this assay. Figure 3B is the particle size increase of the assay solution after anti-VEGF antibody addition. To our surprise, the healthy control mice showed much larger particle size increase than the tumor-bearing mice: the average particle size increase was about 18 nm for healthy control mice and the average particle size increase was only 6 nm for tumor-bearing mice. Furthermore, the particle size increase varied significantly from 8 nm to more than 30 nm among the individual healthy control mice, while the particle size increase varied only from 4 to 9 nm for tumor-bearing mice.

### Human serum protein adsorption to AuNPs and VEGF analysis

Three groups of human serum samples were included in this study: normal healthy donors (n = 15); patients diagnosed with benign prostate hyperplasia (BPH, n = 10); and patients diagnosed with prostate cancer with stages from T1c to T3b (n = 25). A list of the sources and clinical information can be found in the Additional File 1, Table 1S. It should be mentioned here that the healthy donors were asymptomatic, but not clinically confirmed as being cancer-free or without BPH.

We first conducted the analysis of human serum adsorption to the AuNPs. Assaying undiluted human serum samples, no difference between the three groups of human serum samples was observed: the average particle size increase for cancer and the normal/BPH group were essentially equal (Additional file 1, Figure S2). However, there were significant differences between individual samples, suggesting that each individual has different serum molecular profiles. When the serum was diluted 10-fold, the adsorption of all samples (a total of 50 samples from the three groups), caused a uniform particle size increase of around 20-25 nm. Similar to the diluted mice serum protein adsorption study, this size increase was observed within one or two minutes of incubation and remained unchanged thereafter.

**Figure 4:**
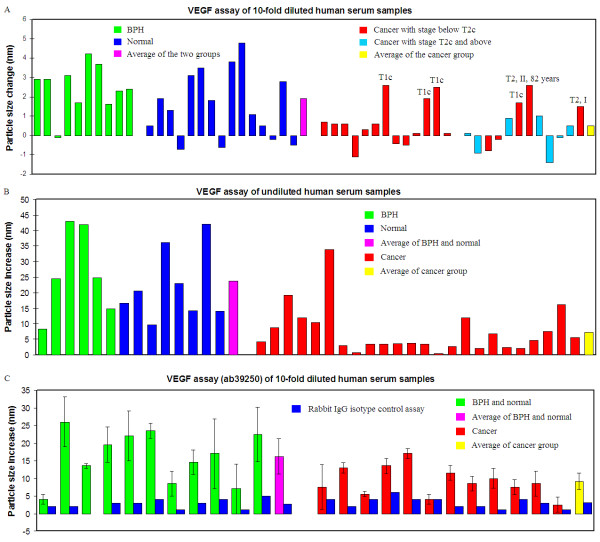
**VEGF assay of human serum samples and control study**. (A) 10-fold diluted and (B) undiluted human serum samples using anti-VEGF antibody (ab9570). The particle size change presented in both graphs is the difference between the size measured at 5 min after antibody addition and the size measured just before antibody addition. (C) 10-fold diluted human serum samples using anti-VEGF antibody (ab39250). The particle size change presented in the graph is the difference between the size measured before antibody addition and the size measured at 9 min after antibody addition. Also shown in (C) is the particle increase of the assay solution by adding a Rabbit IgG isotype control to the assay solution (data in blue colored columns). The particle size change presented in the graph is the difference between the size measured before antibody addition and the size measured at 9 min after antibody addition. Data presented in (A) and (B) were obtained using Malvern ZS90 DLS system; while data presented in (C) were obtained from Nano Discovery NDS-1200 system. Error bar shown in this graph is the standard deviation of two replicate assays.

For VEGF analysis, two anti-VEGF antibodies were used in this study: ab9570 and ab39250. VEGF has several isoforms that are known to play different roles in tumor angiogenesis: VEGF121, 145, 165, 183, 189 and 206 (the number represents the amino acid length of the protein) [26-28]. While ab9570 is specific to VEGF121 and 165, ab39250 is reactive to VEGF121, 165 and 189 [29]. The results shown in Figure 4A and B were obtained from using ab9570 anti-VEGF in the assay, and the results in Figure 4C were obtained from using ab39250 anti-VEGF in the assay.

The VEGF assay was first conducted on the 10-fold diluted human serum samples (Figure 4A) using the ab9570 antibody. Although the particle size change was small, nevertheless, there was a clear difference between the prostate cancer and non-cancerous group. While the average particle size change for the normal and BPH group was about 2 nm, the size change was only ~0.5 nm for the 25 prostate cancer samples. T-test analysis revealed a p-value of 0.001 for this assay. Hence, the difference observed from the cancer and non-cancer group is statistically significant. Furthermore, it was noticed that the samples that exhibited a relatively large particle size increase in the cancer group were all from patients with very early stage cancer (marked in Figure 4A). Among the six samples highlighted, four were from T1c stage cancer and two from T2 stage cancer. Among the two T2 stage samples, one was from a patient 82 years old (most other donors were below 75 years). Another sample, although it was staged as a T2 stage cancer according to the TNM system, it was assigned as a group stage I cancer clinically, indicating that it was a very early stage cancer. By excluding these six early stage cancer samples, the average particle size change of the cancer group became zero. Notably, among all of the cancer samples tested in this study, none of the relatively advanced cancer samples with a stage from T2c to T3b (total 7, data marked in cyan) showed a particle size increase of more than 1 nm.

This same trend was also observed in the undiluted serum samples (Figure 4B) using the same antibody as used in Figure 4A. While the average particle size increased by 24 nm for the non-cancerous group, the average particle size increased only by 7 nm for the prostate cancer samples. T-test analysis of this set of assay data gave a p-value of 0.001. Compared to the 10-fold diluted serum samples, the VEGF assay of undiluted serum resulted in a substantial particle size increase, indicating that without dilution, more VEGF proteins were detected from the AuNP surface.

Although the VEGF assay of both diluted and undiluted samples revealed statistically significant differences between cancerous and non-cancerous samples, there are some issues with the above assays. For the diluted serum samples, the particle size increase caused by the anti-VEGF addition is rather small and too close to the experimental error of the DLS measurement, which is 1-2 nm for the AuNP probe solution. For the undiluted serum samples, the initial particle size after serum protein corona formation varies significantly from one sample to another as seen from Additional file 1, Figure S2. This makes it difficult to compare the nanoparticle size increase caused by the anti-VEGF antibody addition. It also makes it difficult to use such particle size increases to evaluate the different levels of VEGF in the protein corona. In an attempt to optimize the assay, we used a different anti-VEGF antibody for the analysis. Figure 4C is the VEGF assay results of 10-fold diluted human serum samples obtained using the ab39250 antibody. The same trend was observed as in the other assay using the ab9570 antibody. Non-cancerous serum samples showed a higher level of VEGF in the serum protein corona adsorbed on AuNPs than the cancer group samples. The average particle size increase was 16 nm for the non-cancer group and 9 nm for the cancer group samples. The p-value for this assay is 0.009, indicating that the observed difference is statistically significant.

**Figure 5:**
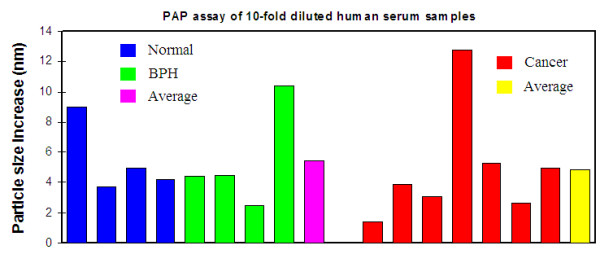
**PAP assay of 10-fold diluted human serum samples**. Normal and BPH samples were randomly selected from the total 25 samples. Cancer samples used for this assay are those with a stage of T2c or above. The particle size change is the difference between the size measured at 5 min after antibody addition and the size measured just before antibody addition.

For comparison, we also analyzed an established prostate cancer biomarker, prostatic acid phosphatase (PAP) [30], in this study. The assay results (Figure 5) showed no difference between the cancer and non-cancer samples: the average particle size increase observed from the normal and BPH group was almost the same as the cancer group. The p-value of this assay was 0.73. Even though PAP is known as a biomarker for prostate cancer, it is not a biomarker for early stage prostate cancer detection. It only shows a significant difference when prostate cancer has advanced into the metastasized stage [30]. None of the samples tested in our study is from a metastasized prostate cancer. Therefore, it is not surprising that we did not observe difference between cancer and non-cancer samples in PAP assay.

### Specificity study of the VEGF assay of human serum samples

To confirm that the particle size increase after anti-VEGF antibody addition to the assay solution is indeed due to the specific binding between the antibody and VEGF protein adsorbed on AuNPs, we conducted two control experiments. The first experiment was to use a non-specific isotype control, Rabbit IgG, to repeat the assay. The results are included in Figure 4C along with the anti-VEGF assay results for comparison. It is clear that the particle size increase caused by anti-VEGF addition was substantially larger than the isotype control antibody: 16 nm for the non-cancer group and 9 nm for the cancer group. The average particle size increase caused by Rabbit IgG addition was only around 2.7 nm. More importantly, there is no difference between the average particle size increase of the cancer versus non-cancerous group, confirming that Rabbit IgG binds only weakly and non-specifically to the serum protein-adsorbed AuNPs.

**Figure 6:**
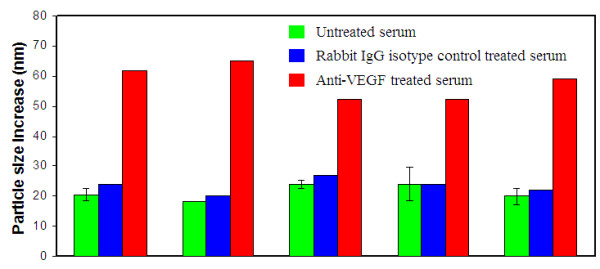
**Antibody-treated and untreated serum adsorption study to AuNP**. Three types of samples were analyzed: 10-fold diluted human serum; Rabbit IgG isotype control-treated 10-fold diluted human serum; and anti-VEGF (ab39250) treated 10-fold diluted human serum samples. Five samples that show the highest particle size increase from the anti-VEGF assay as shown in Figure 4C were chosen for this study.

In a second control experiment, we chose 5 diluted serum samples (four from the normal group and one from the cancer group) that show the highest particle size increase from the anti-VEGF assay as shown in Figure 4C. We then added an equal amount of Rabbit IgG and anti-VEGF (ab39250) to an equivalent volume of the diluted serum samples (i.e. 5 μL of 10-fold diluted serum with 5 μL of Rabbit IgG or anti-VEGF at 1 mg/mL) and incubated the mixed solution for 30 minutes at r.t. The treated samples (4 μL) were then mixed with AuNP solution (40 μL), and the particle size increase of the assay solution was analyzed. Figure 6 is the average particle size increase observed from the untreated diluted serum, Rabbit IgG-treated diluted serum, and anti-VEGF-treated diluted serum samples, respectively. Without the pretreatment of any antibody, the average particle size of the assay solutions increased by about 20-25 nm, the same as we observed throughout the study. When the serum samples were pretreated with the non-specific Rabbit IgG isotype control, the particle size increase was about the same as that without antibody addition. This suggests that Rabbit IgG exhibited minimum binding to the serum proteins, and had little effect on the size of the "protein corona" formed on the AuNP surface. In contrast to the Rabbit IgG isotype control, the addition of anti-VEGF to the diluted serum caused a significantly larger particle size increase of more than 50 nm. This result confirmed the specific binding between anti-VEGF and VEGF proteins in the serum samples.

## Discussion

This study has led to several very interesting findings. Of all the results, the first most interesting finding is that there is a significant difference in the "size" of the serum protein corona formed on the AuNPs surface between cancerous and non-cancerous, or less malignant tumor-bearing mice models. Although the exact molecular mechanism behind these differences is unclear, we cautiously propose that the different protein corona sizes are reflective of the different "complexing level" of serum proteins in the samples, not due to unsaturated coverage of proteins on the AuNPs. As explained at the beginning of the "Results" section, even after the serum is diluted 10-times, the total serum protein concentration is still 10^4^-10^5 ^times higher than the AuNP concentration. In previous work we have conducted, we found that most IgG molecules will form a complete coverage on the AuNPs in just one or two minutes at a concentration between 0.1-1 mg/mL. Even in the 10-fold diluted serum samples, the IgG concentration is still at the 1 mg/mL range. IgG molecules alone in the diluted serum samples should be able to form a complete coverage on the AuNPs surface. The substantially smaller protein corona found from PC3 mice compared to the other two groups suggests that when tumor progresses, there may be some chemicals released from the tumor cells that have led to the disruption of serum protein complexing. The relative ratio of tumor versus body weight of PC3 mice (5%) is enormous compared to LnCaP mice (0.3%) and typical human cancer patients. Because of the large tumor grown in PC3 mice, the molecular change is manifest and all PC3 mice serum samples exhibit a uniformly small protein corona in the assay.

In addition to the difference between different mice groups, it was also noticed that the protein corona size varies substantially between individual mice within the same group, particularly in the LnCaP mice and healthy control group. All mice were the same type and same age (the mice were randomized into the three groups before experiments), and were kept at the same environment under the same diet during the experiment. The difference observed within the same mice group can only be attributed to the different biological responses and physical conditions of individual mice. Cancer is well known for its heterogeneity and for its extremely complicated and diverse molecular mechanisms. The molecular differences observed from our study appear to corroborate with this well known fact and suggest the need of personalized medicine for cancer treatment.

From the human serum study, we did not observe significant difference in the serum protein corona size between cancer and non-cancer groups. All human cancer serum samples used in this study are from a relatively early stage prostate cancer of T1c to T3b, and the cancer has not yet metastasized. Because of the relatively small tumor size, the molecular change in the serum caused by cancer is not as obvious as that found from the mice model study.

The second most significant finding observed from this study is the different VEGF levels that were found in the mice and human serum samples. The VEGF assay of both human and mouse serum samples revealed that in the protein corona adsorbed to AuNPs, there is a decreased level of VEGF in the cancerous samples as compared to the samples from healthy and benign conditions. This finding appears to be contradictory to what is generally believed about VEGF: that the level of VEGF is often elevated in the blood of cancer patients [31-33]. We attribute this discrepancy to the following possible reason. VEGF is not an abundant protein in the serum. Its concentration in blood is commonly reported to be in the high pg/mL to low ng/mL range [32,33]. This concentration is 10^6^-10^9 ^times lower than the abundant serum proteins such as IgG and serum albumin, which occur in a concentration range of 10s mg/mL. Upon mixing with AuNPs, abundant proteins such as IgG and serum albumin will be quickly adsorbed to AuNPs. It is most likely that the VEGFs detected in the assay are adsorbed to the AuNPs through a more abundant "carrier" protein or other types of biomolecules. In other words, the VEGF detected from our assay is a serum protein-complexed form of VEGF, not the typical individual VEGF proteins as detected using other existing immunoassay methods.

In addition to the above two most important findings, the IgG assay of mouse serum samples is also very interesting. Mice with small tumor grown from LnCaP cells showed substantially higher IgG level than normal healthy mice and mice with large tumor grown from PC3 cells (Figure 3A). This difference suggests that mice responded differently to the orthotopic injection of LnCaP and PC3 cells. It appears that the injection of LnCaP cells may have triggered certain immunogenic response from the mice, and such immunogenic response has slowed down the tumor growth. The significant variation of IgG level within the same mice group is again most likely due to the different biological responses and physical conditions of individual mice.

## Conclusions

In summary, we reported here a facile nanoparticle-based immunoassay for serum protein analysis. Serum is a complex biological fluid containing trace and abundant proteins, macromolecular complexes and microparticles that can cause significant interference to light scattering technique-based assays. Hereto, by taking advantage of the exceptionally strong scattering intensity of gold nanoparticles, we were successful in overcoming this challenge and developed a homogeneous solution assay for serum protein analysis. Using this simple assay, we observed multiple molecular differences between prostate cancer and non-cancerous samples. Analysis of samples from both mouse models and human subjects revealed that the amount of AuNP-adsorbed VEGF is decreased in cancer samples. We are currently conducting further extensive studies to understand the mechanistic origins of these molecular changes. By screening other proteins or biomolecular targets adsorbed to the AuNPs, it is likely that additional proteins or biomolecular profiles that are unique to disease states will be discovered. The assay method reported here can potentially become a general new approach for cancer biomarker discovery and research.

## Methods

### Chemical and Biochemicals

Gold nanoparticle (AuNPs) with a diameter of 90 nm as measured by DLS (15708-9) was purchased from Ted Pella Inc. (Redding, CA). The concentration of this gold nanoparticle was 10 pM. All the antibodies used in the present study were from Abcam (Cambridge, MA): rabbit polyclonal anti-mouse IgG (ab6708, lot 951580, 2.0 mg/mL); rabbit polyclonal anti-VEGF (ab9570, lot 942538, 0.5 mg/mL); rabbit polyclonal anti-VEGF (ab39250, lot GR22131-1, 1 mg/mL); rabbit polyclonal anti-prostatic acid phosphatase (PAP) (ab97517, lot gr742-1, 0.8 mg/mL); and rabbit IgG isotype control (ab37415, lot 905012, 5 mg/mL).

### Animal and human serum samples

Mouse serum samples used in this study were obtained from mice models prepared according to procedures described in the Additional File 1. This study was approved by University of Central Florida Institutional Animal Care and Usage Committee (IACUC) under the protocol number of 09-26. Specific pathogen-free conditions and facilities, approved by the American Association for Accreditation of Laboratory Animal Care (AAALAC) and compliant with the regulations and standards of the United States Department of Agriculture, the United States Department of Health and Human Services and the NIH, were used to house and maintain all mice.

Human serum samples from male donors diagnosed with prostate cancer or benign prostate hyperplasia, and healthy controls were obtained from MD Anderson Cancer Center Orlando (MDACCO) and Asterand Solutions (solutions.asterand.com). The collection and use of human serum samples for the project was approved by MDACCO IRB committee under the protocol number of 09.061.09. Human serum samples from Asterand Solutions were de-identified archived samples. No IRB approval was required for using these samples. A list of detailed information on the source and clinical data of human serum samples can be found in the Additional file 1, Table S1.

### DLS measurements

The DLS measurements were conducted using two different instruments: The first one is a Zetasizer Nano ZS90 DLS system equipped with a red laser (633 nm) and an Avalanche photodiode detector (APD) (Malvern Instruments Ltd, England). The second instrument is an automatic NDS-1200 DLS system from Nano Discovery Inc. The detection system of NDS-1200 is similar to the ZS90 system. Additionally, NDS-1200 is equipped with a 12 sample holder-carousel that allows automatic measurement of 12 samples within 5-6 minutes. Data presented from Figure 2 to Figure 4A and B, and Figure 5 were obtained from ZS90 system, and data presented in Figure 4C and Figure 6 were from NDS-1200 system. The average particle size of the solution was obtained using a Cumulant method for both systems.

### Assay methods

In the serum adsorption study, 40 μL of AuNP solution was mixed with 2 μL of undiluted or 10-fold diluted serum sample in 10 mM phosphate buffer (PB). The particle size of the mixed solution was measured three times at 1, 5, and 8 min of incubation for the undiluted serum samples, and twice at 1 and 5 min of incubation for the diluted serum samples. Data presented in Figure 2A are the particle size difference measured at 8 min and 1 min of incubation; and data presented in Figure 2B are the particle size difference measured at 5 min and 1 min of incubation. For both undiluted and diluted mouse serum protein adsorption study, each assay was repeated twice and the error bar presented in Figure 2A and 2B are the standard deviations. Results of serum protein adsorption study of undiluted human serum samples are presented in Additional file 1, Figure S2. The cancer group samples were assayed twice and the error bars are standard deviations. When the serum was diluted 10 fold, the adsorption of all diluted human serum samples, observed from total 50 samples from the three different groups, caused a uniform particle size increase of around 20-25 nm.

For target protein assays (IgG, VEGF or PAP), an appropriate amount of antibody solution was added to the assay solution following the serum adsorption to AuNPs. The particle size change was then measured again by DLS after 5 minutes of incubation. The amount of the antibody solution added to the serum-AuNP mixture solution was: 1 μL for anti-mouse IgG; 2 μL for anti-VEGF; and 2 μL for anti-PAP. Different volumes of antibody solution were used in different assays to ensure that the amount of the antibody used for each assay is about the same. For mouse IgG assay, 6 samples randomly selected from the three sample groups were assayed twice and the error bars shown in Figure 2A are standard deviations.

All incubation and particle size analysis were done at ambient temperature of 25°C.

### Statistical Analysis

We applied T-tests for comparison of means of human serum sample assay results. These tests assume that the data are normally distributed and that individuals in different groups are independent. We tested the null hypothesis that the means of two groups (normal and BPH were combined as one group and prostate cancer as the second group) are equal versus the alternative that they are not equal, with and without the assumption of equal variances in the two groups. Statistical analysis resulted in p-values for each of the individual tests. Small p-values can be viewed as strong evidence that the differences between the two sample groups are statistically significant.

## Competing interests

QH is a shareholder and officer of Nano Discovery Inc (Orlando, FL) which has a potential financial interest in this work. Nano Discovery Inc licensed the NanoDLSay™ technology as cited in this paper from University of Central Florida for commercial usage. All other authors have no competing interest.

## Authors' contributions

QH designed the research, conducted experiments, data analysis, and prepared the manuscript. JC prepared the animal model and collected mice serum samples for this study, and also participated in the manuscript preparation. AC and JB conducted part of the assay experiments. CHB supervised the animal model study and human serum collection, and also participated in the manuscript preparation. SG provided scientific insights into the experimental design and interpretation of the data, and participated in the manuscript preparation. MYP conducted the statistical analysis of the study. All authors have read and approved the final manuscript.

## Supplementary Material

Additional file 1 **Experimental details on animal model development and sample collection; a list of human serum samples used in the present study with source and clinical information; and the results of human serum adsorption to AuNPs can be found in Additional File **1. Click here for file
