# Supplementary material for: A Facile Nanoparticle Immunoassay for Cancer Biomarker Discovery
Source: J Nanobiotechnology. 2011 May 23;9:20. doi: 10.1186/1477-3155-9-20 (PMC3127990; doi:10.1186/1477-3155-9-20)
Supplement: Additional file 1 — Experimental details on animal model development and sample collection; a list of human serum samples used in the present study with source and clinical information; and the results of human serum adsorption to AuNPs can be found in Additional File 1. [file 1477-3155-9-20-S1.DOC]

**Supplementary Information**

**A Facile Nanoparticle Immunoassay for Cancer Biomarker Discovery**

Qun Huo,^1*^ Jimmie Colon,^2^ Adam Cordero,^1^ Jelena Bogdanovic,^1^ Cheryl H. Baker,^2^ Steven Goodison,^2^ Marianna Y. Pensky^3^

^1^NanoScience Technology Center and Department of Chemistry, University of Central Florida, 12424 Research Parkway Suite 400, Orlando, FL 32826

^2^MD Anderson Cancer Center Orlando, 6900 Lake Nona Blvd, Orlando, FL 32827

^3^Department of Mathematics, University of Central Florida, 4000 Central Florida Blvd, Orlando, FL 32816

*To whom the correspondence should be addressed: [qhuo@mail.ucf.edu](mailto:qhuo@mail.ucf.edu) Tel: 407-882-2845

**1. Establishment of human prostate tumors in mice and sample collection**

*1.1 Cell Culture:* The human prostate cancer cell lines PC3 (CRL 1435) and LnCaP (CRL 1740) (ATCC, Manassas, VA) were maintained in F-12K and RPMI- 1640 media, respectively The cell culture media was supplemented with 10% fetal bovine serum (FBS) and 1% penicillin-streptomycin (Invitrogen, Carlsbad, CA). Adherent monolayer cultures were maintained at 37°C in 5% CO_2_.

***1.2 Orthotopic injection of prostate cancer cells in athymic nude mice:*** Six- to eight-week-old male athymic nude mice (NCI-*nu*) were purchased from the Animal Production Area of the National Cancer Institute (NCI) Frederick Cancer Research and Development Center (Frederick, MD). Specific pathogen-free conditions and facilities, approved by the American Association for Accreditation of Laboratory Animal Care (AAALAC) and compliant with the regulations and standards of the United States Department of Agriculture, the United States Department of Health and Human Services and the NIH, were used to house and maintain all mice. Animals were supplied with ¼” cob bedding (Harlan), irradiated food (Harlan Teklad 2919) and autoclaved tap water. To produce tumors, PC3 and LnCap cells were harvested from subconfluent cultures by a brief exposure to 0.25% trypsin and 0.02% EDTA. Trypsinization was stopped with medium containing 10% fetal bovine serum, and the cells were washed once in serum-free medium and resuspended in HBSS. Only suspensions consisting of single cells with >90% viability were used for the injections.


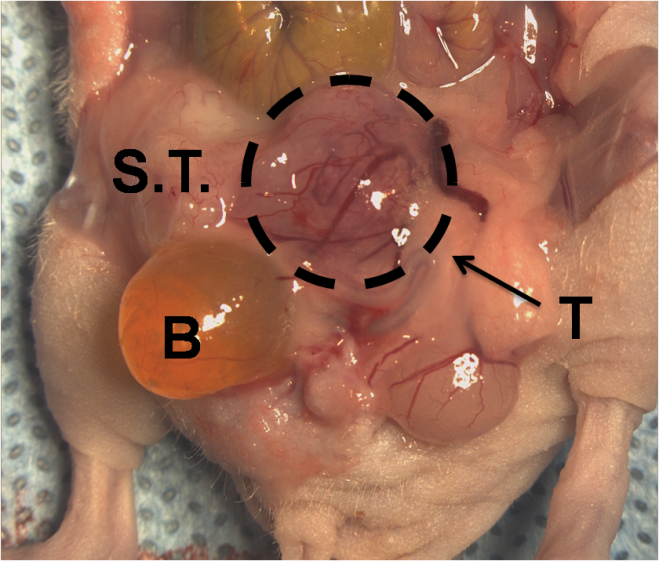
The mice were randomized into three groups as follows: (a) prostate injection of 1X PBS in control groups (n=5), (b) 250,000 PC3 (n=5) or LnCap (n=6) cells into the prostate. Under proper anesthesia, a lower midline incision was made to the mice to expose the prostate. Two hundred and fifty thousand (250,000) PC3 or LnCap cells (in 10 µl of cell culture media) were injected using a 30-gauge hypodermic needle on a 100 µL Hamilton Syringe (VWR International, Suwanee, GA) using a Leica MZ-16 dissecting microscope (MicroOptics of Florida, Davie, FL). Surgical clips were used for primary wound closure and weekly monitoring of body weight was performed.

**Figure S1**. Mouse orthotopic model of human prostate cancer. Shown here are the primary prostate tumor (T), the seminal tubes (S.T.), and the bladder (B).

*1.3 Tumor and blood sample collection:* Mice were killed when moribund (4-5 wk after injection). Tumors were harvested and serum was collected on the same day. For mice injected with PC3 cells, mouse serum was collected on day 28. For mice injected with LnCap cells and control PBS solution, mouse serum was collected on days 35. Blood was collected by cardiac puncture. The primary prostate tumors (T) were surgically separated from the seminal tubes (S.T.) and the bladder (B) (Figure 1S), and the tumor size and weight were recorded. Tumor volumes were calculated by using the following formula: 0.5 x (length) x (width)^2^.

**2. Serum protein adsorption study of undiluted human serum samples**


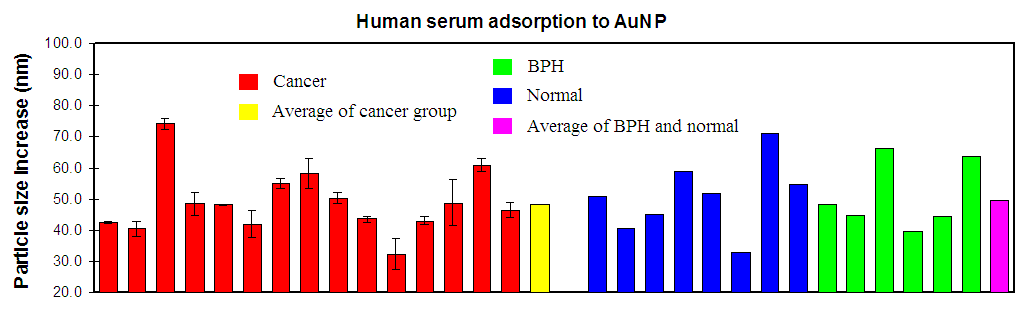


**Figure S2.** Nanoparticle size increase after adsorption of undiluted human serum samples. Particle size increase presented in the graph is the difference between particle size measured at 8 minute of incubation and 1 min of incubation. Each sample from the cancer group was assayed twice and the error bars are standard deviations of the measurements.

**Table S1. Information of human serum samples used in the study^*^**

| **sample number** | **clinical** | **age** | **PSA** | **Collection date** | **Collection Site** | **Receiving date** |
| --- | --- | --- | --- | --- | --- | --- |
| 43332 | BPH | 78 |  | 2005 | AST19-EastEu | 8/25/2009 |
| 43189 | BPH | 79 |  | 2005 | AST19-EastEu | 8/25/2009 |
| 43159 | BPH | 64 |  | 2005 | AST19-EastEu | 8/25/2009 |
| 43160 | BPH | 64 |  | 2005 | AST19-EastEu | 8/25/2009 |
| 43104 | BPH | 76 |  | 2005 | AST19-EastEu | 8/25/2009 |
| 43029 | BPH | 74 |  | 2005 | AST19-EastEu | 8/25/2009 |
| 145196 | BPH | 70 |  | 2009 | AST53-US | 9/30/2010 |
| 158130 | BPH | 79 |  | 2010 | AST165-US | 9/30/2010 |
| 147824 | BPH | 71 |  | 2009 | AST68-US | 9/30/2010 |
| 179892 | BPH | 73 |  | 2010 | AST52-US | 9/30/2010 |
|  |  |  |  |  |  |  |
| 132832 | normal | 40 |  | 2009 | AST54-US | 8/25/2009 |
| 132803 | normal | 40 |  | 2009 | AST54-US | 8/25/2009 |
| 132846 | normal | 41 |  | 2009 | AST54-US | 8/25/2009 |
| 132709 | normal | 37 |  | 2009 | AST54-US | 8/25/2009 |
| 130854 | normal | 37 |  | 2009 | AST54-US | 8/25/2009 |
| 130863 | normal | 37 |  | 2009 | AST54-US | 8/25/2009 |
| 130859 | normal | 35 |  | 2009 | AST54-US | 8/25/2009 |
| 130869 | normal | 38 |  | 2009 | AST54-US | 8/25/2009 |
| 121229 | normal | 37 |  | 2008 | AST54-US | 9/30/2010 |
| 121180 | normal | 35 |  | 2008 | AST54-US | 9/30/2010 |
| 121172 | normal | 36 |  | 2008 | AST54-US | 9/30/2010 |
| 130877 | normal | 35 |  | 2009 | AST54-US | 9/30/2010 |
| 145156 | normal | 35 |  | 2009 | AST54-US | 9/30/2010 |
| 145342 | normal | 40 |  | 2009 | AST54-US | 9/30/2010 |
| 173797 | normal | 31 |  | 2010 | AST52-US | 9/30/2010 |
|  |  |  |  |  |  |  |
| N001A | T2aN0M0, 6 | 64 | 3.3 | 9/14/2009 | MDACCO | 2/19/2010 |
| N002A | T1cN0M0, 7 | 73 | 4.3 | 9/15/2009 | MDACCO | 2/19/2010 |
| N004A | T2aN0Mo, 6 | 73 | 4.2 | 10/27/2009 | MDACCO | 2/19/2010 |
| N005A | T1cN0M0, 8 | 72 | 8.9 | 11/10/2009 | MDACCO | 2/19/2010 |
| N008A | T1cN0M0, 7 | 59 | 4.1 | 2/1/2010 | MDACCO | 2/19/2010 |
| N009A | T2aN0M0, 6 | 69 | 2 | 2/3/2010 | MDACCO | 2/19/2010 |
| N014A | T1cN0M0, 7 | 59 | 8.3 | 2/23/2010 | MDACCO | 6/24/2010 |
| N015A | T2aN0M0, 7 | 73 | 2.5 | 3/15/2010 | MDACCO | 6/24/2010 |
| N016A | T2aN0M0,7 | 65 | 7 | 3/23/2010 | MDACCO | 6/24/2010 |
| N017A | T1cN0M0, 7 | 74 | 6.6 | 4/6/2010 | MDACCO | 6/24/2010 |
| N018A | T1cN0M0, 6 | 56 | 5.2 | 4/7/2010 | MDACCO | 6/24/2010 |
| N019A | T1cN0M0, 7 | 72 | 6 | 4/12/2010 | MDACCO | 6/24/2010 |
| N023A | T1cN0M0, 6 | 59 | 8.1 | 9/3/2010 | MDACCO | 9/3/2010 |
|  |  |  |  |  |  |  |
| 128826 | T2cN0M0, II | 50 | 7.9 | 2010 | AST8-US | 8/11/2010 |
| 125823 | T3bN0Mx, III | 61 | 4.9 | 2010 | AST8-US | 8/11/2010 |
| 125833 | T2N0M0, I | 55 | 1.9 | 2010 | AST8-US | 8/11/2010 |
| 167077 | T2N0M0, II | 63 |  | 2010 | AST74-US | 8/11/2010 |
| 158140 | T3bN0M0, III | 62 |  | 2010 | AST165-US | 8/11/2010 |
| 173814 | T1cNxMx, I | 65 |  | 2010 | AST165-US | 8/11/2010 |
| 130817 | T2NxMx, II | 82 |  | 2009 | AST165-US | 8/11/2010 |
| 130835 | T3aN0Mx, IV | 72 | 0.01 | 2009 | AST165-US | 8/11/2010 |
| 127274 | T3bNxMx, III | 47 | 4.4 | 2009 | AST8-US | 8/11/2010 |
| 125920 | T2cNxMx, II | 69 | 1.2 | 2009 | AST8-US | 8/11/2010 |
| 125848 | T3bN0Mx, III | 74 | 7.73 | 2009 | AST8-US | 8/11/2010 |
| 153482 | T2N0M0, I | 70 |  | 2009 | AST134-US | 8/11/2010 |

* All human serum samples, except those from MDACCO (MD Anderson Cancer Center Orlando), were obtained from Asterand Solutions, Inc (solutions.asterand.com). For samples collected from MDACCO, appropriate IRB approval was obtained through the Institute. For samples obtained from Asterand Solutions, samples were collected from different clinics in US and East Europe. All samples were de-identified before they were received by the Principal Investigator’s laboratory for analysis. All samples were stored at -80 ^o^C before shipping or transfer. Upon receiving the samples, they were aliquoted into appropriate volume and stored at -20 ^o^C prior to analysis.
